# Structural complexities and sodium-ion diffusion in the intercalates Na_*x*_TiS_2_: move it, change it, re-diffract it†

**DOI:** 10.1039/c9ra05690d

**Published:** 2019-09-03

**Authors:** Dennis Wiedemann, Emmanuelle Suard, Martin Lerch

**Affiliations:** Technische Universität Berlin, Institut für Chemie 10623 Berlin Germany dennis.wiedemann@chem.tu-berlin.de; Institut Laue-Langevin 38042 Grenoble France

## Abstract

After momentary attention as potential battery materials during the 1980s, sodium titanium disulphides, like the whole Na–Ti–S system, have only been investigated in a slapdash fashion. While they pop up in current reviews on the very subject time and again, little is known about their actual crystal-structural features and sodium-ion diffusion within them. Herein, we present a short summary of literature on the Na–Ti–S system, a new synthesis route to Na_0.5_TiS_2_-3*R*_1_, and results of high-temperature X-ray and neutron diffractometry on this polytype, which is stable for medium sodium content. Based thereon, we propose a revision of the crystal structure reported in earlier literature (missed inversion symmetry). Analyses of framework topology, probability-density functions, and maps of the scattering-length density reconstructed using maximum-entropy methods (all derived from neutron diffraction) reveal a honeycomb-like conduction pattern with linear pathways between adjacent sodium positions; one-particle potentials indicate associated activation barriers of *ca.* 0.1 eV or less. These findings are complemented by elemental analyses and comments on the high-temperature polytype Na_0.9_TiS_2_-2*H*. Our study helps to get a grip on structural complexity in the intercalates Na_*x*_TiS_2_, caused by the interplay of layer stacking and Na–Ti–vacancy ordering, and provides first experimental results on pathways and barriers of sodium-ion migration.

## Abstract 2

The structure of Na_*x*_TiS_2_-3*R*_1_ was revised and honeycomb-like pathways with their barriers of sodium-ion migration were found based on high-temperature neutron diffraction.

## Graphical abstract

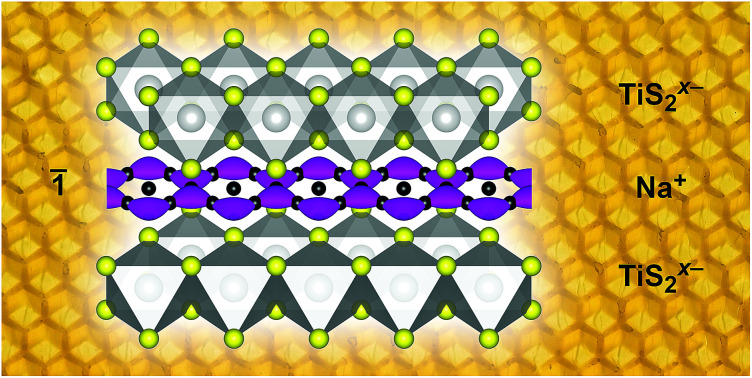

## Introduction

Layered lithium-ion conductors are a successful substance class for energy storage, *e.g.*, in modern consumer electronics. Much effort has been put into research on their sodium congeners as possible high-voltage, low-cost alternatives—so far, to no commercial avail. Although alkali transition-metal dichalcogenides are not a recent focus, they pop up in current reviews time and again.^1^ Probably as analogues of the once front-running lithium intercalates Li_*x*_TiS_2_, the sodium compounds Na_*x*_TiS_2_ received some attention in the early to mid-1980s, leading to an overall manageable corpus of literature on the Na–Ti–S system. They have also sparked some commercial interest, manifested in patents, as thermoelectrics and auxiliary materials for cathodes in sodium-ion batteries.^2,3^

**Fig. 1:**
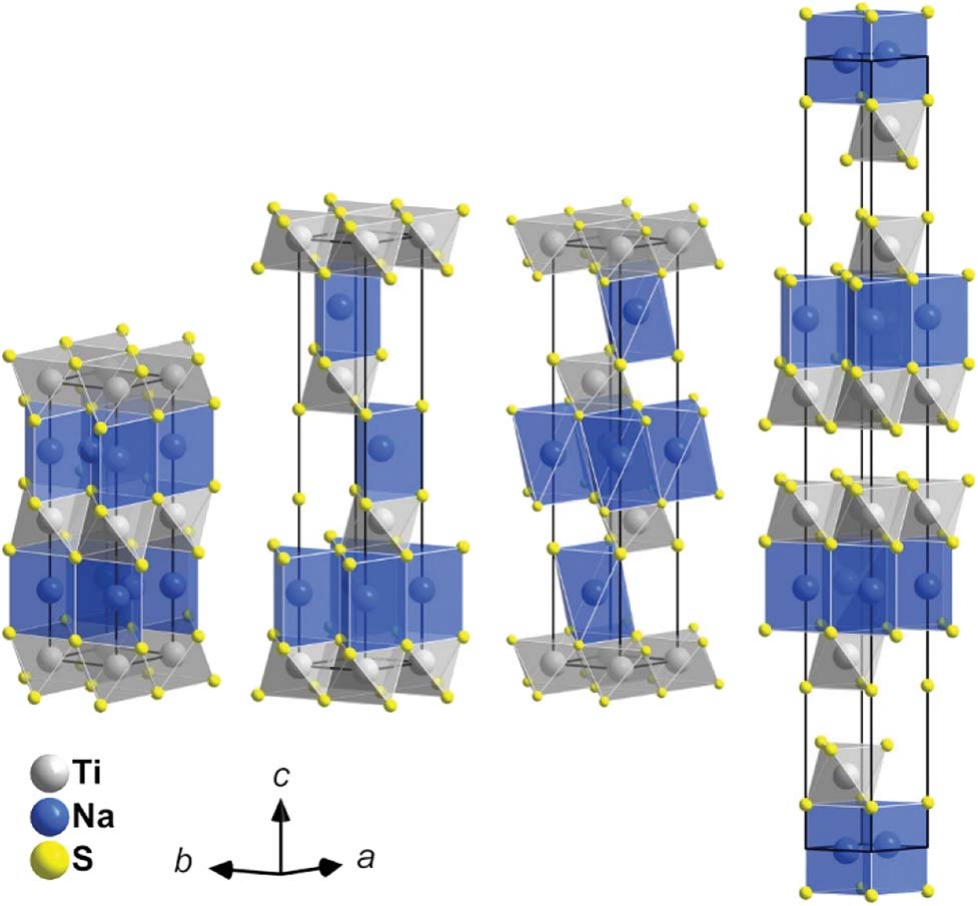
Crystal structures of the Na_*x*_TiS_2_ polytypes; left to right: 2*H*, 3*R*_1_, 3*R*_2_, and 6*H*. Atoms are plotted with arbitrary radii, unit cells are depicted in black. Note: depending on *x*, sodium positions may not be fully occupied.

As first Na–Ti–S compound, uncharacterised “Na_2_TiS_3_” was reported in a process patent to have an X-ray pattern similar to the also uncharacterised “Li_2_TiS_3_” and “K_2_TiS_3_” in 1960.^4^ Five years later, Rüdorff described the first layered structure in the system and wrongly assigned it the NaHF_2_ type [modern prototype according to the Inorganic Crystal Structure Database (ICSD): CrOOH(*R*3̄*m*)]—not yet aware of its hydrogen positions.^5^ Reliable sodium positions were not established before the first systematic study in 1971.^6^ Today, five kinds of Na–Ti–S compounds can be distinguished:

• The structure of the intercalates Na_*x*_TiS_2_ with 0 < *x* ≤ 1 consists of ^2^_∞_[TiS_2_]^*x*−^ sandwich layers with sodium ions in between. The compounds exist in four different structurally elucidated stacking variants: the polytypes 2*H*, 3*R*_1_, 3*R*_2_, and 6*H* (see Table 1 and Fig. 1). With these materials, the remaining article is concerned.

**Table 1:** Crystal-structurally characterised polytypes of Na_*x*_TiS_2_ (0 < *x* ≤ 1) from literature

Polytype		2*H*	3*R*_1_	3*R*_2_	6*R*
Synonyms		2H(I)	3R(I), phase I *b*	3R(II), phase I *a*	6R(I), phase II
Stability range^a^		High-temperature phase	0.40(2) < *x* < 0.64(2) (0.35 < *x* < 0.72)	0.75(2) < *x* ≤ 1 (0.70 < *x* ≤ 1)	0.15(2) < *x* < 0.25(2) (0.10 < *x* < 0.33)
Best characterised representative		*x* = 1 (ref. 7)	*x* = 0.55 (ref. 6)	*x* = 1 (ref. 6)	*x* = 0.3 (ref. 6 and 8)
Coordination polyhedron^9^	Na	[6*p*]	[6*p*]	[6*ap*]	[6*p*]
	Ti	[6*o*]	[6*o*]	[6*o*]	[6*o*]
Stage		1	1	1	2
Space-group type		*P*6_3_/*mmc*	*R*3*m*^c^	*R*3̄*m*	*R*3̄*m*
Structure prototype (ICSD)		NaTiS_2_	CuCrSe_2_^d^	Delafossite-NaCrS_2_	Ag_1.5_Nb_6_S_12_
Delmas' notation^10^		P2	P3	O3	O1–P3^e^
Anion stacking		AABB	AABBCC	ABCABC	ABABBCBCCACA
Sodium-ion order^b^		Disordered over all voids	Ordered in half of the voids	Ordered in half of the voids	Disordered over all voids

a Best values;^11^ reported maximum extents in parentheses.

b Within occupied sodium layers.

c Corrected for a missed mirror plane,^12^ originally given as *R*3.

d Corrected in May 2019 (see Table S1),^13^ formerly ambiguously given as CuCrSe_2_–AgCrSe_2_(*R*3*m*).

e O1 layers are empty.

• The excess-titanium compounds Na_*x*_Ti_1+*δ*_S_2_ with 0 < *δ* ≤ ⅓ and 0 < *x* ≤ 1 − 3*δ* contain additional titanium ions in intercalation layers, which hinder diffusion of the otherwise mobile sodium ions.^14^

• Contrary to its first assignment, Na_2_TiS_3_ ≡ Na_1.3̄_Ti_0.6̄_S_2_ exhibits a structure homeotypic to Na_2_SnS_3_, not K_2_TiS_3_. At closer inspection, it crystallises in a sixfold superstructure of the 3*R* polytypes with an average structure akin to Na_*x*_TiS_2_-3*R*_2_. It represents an OD structure with alternating pure sodium and mixed sodium-titanium layers, constituting a distorted NaCl homeotype.^15^ Upon heating, Na_2_TiS_3_ decomposes to NaTiS_2_-2*H* under oxidation of sulphide.^8^ The compound dubbed “3*R*′(II)-NaTiS_2_” most probably is a sodium-deficient variant of Na_2_TiS_3_.^8,16^

• The electrochemically intercalated compounds Na_*x*_TiS_3_ contain sulphide as well as disulphide ions and are formed from TiS_3_*via* disulphide (instead of titanium) reduction and concomitant bond cleavage.^17^ They have neither been studied *ex situ* nor structurally characterised.

• Structurally complex Na_4_TiS_4_ has only been reported once in a book of poster abstracts. It is said to crystallise in the orthorhombic space group *Fdd*2 with *a* = 38.49(2), *b* = 59.36(3), *c* = 7.033(2) Å, and *Z* = 120. The unit cell allegedly contains eight crystallographically independent TiS_4_ tetrahedra, which are arranged in triple slabs parallel to (010).^18^

The polytype, which Na_*x*_TiS_2_ assumes, depends on *x* as well as on temperature. In the past, inconsistencies in reported stability ranges were discussed and *bona fide* best values were derived acknowledging synthetic (chemical *vs.* electrochemical intercalation) and analytical differences (diffraction patterns *vs.* discharge curves).^11^ The only reported thermally induced phase transformations are the entropy-driven order–disorder transition for small *x* according to eqn (1) and the irreversible reconstitution of the (at ambient temperature metastable) 2*H* phase for large *x* following eqn (2).^8^ Transformations to the 2*H* form from either of the 3*R* polytypes were not observed and an irreversible transition between the latter was attributed to decomposition *via* sodium loss.1
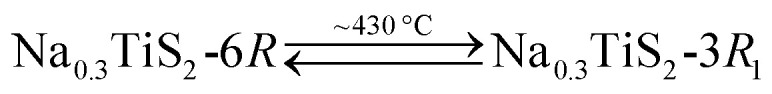
2
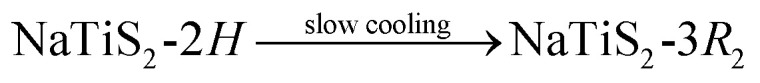


Ordering of sodium ions and vacancies was detected in electrochemically intercalated TiS_2_ single crystals, where 2 × 2, 
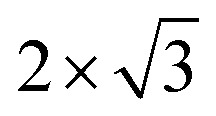
, and 
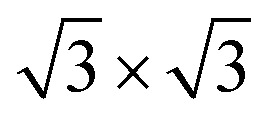
 superstructures may occur. For *x* < 0.11, a stage-3 compound with a 
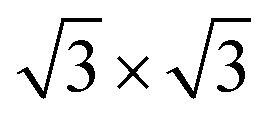
 superstructure of a 3*R* phase containing sodium in trigonal-antiprismatic coordination was discovered but not conclusively characterised.^19,20^ The existence of such superstructures was later rationalised,^21^ comprehensively computed and explained.^22^

Data on sodium-ion diffusion in Na_*x*_TiS_2_ are sparse. The chemical diffusivity at ambient temperature is specified as *ca.* 10^−9^ cm^2^ s^−1^ for 0.25 < *x* < 0.6 (probably polytype 3*R*_1_).^23,24^ The much higher value of 10^−7^ to 10^−6^ cm^2^ s^−1^ for *x* < 0.45, which was only reported once, remains questionable.^25^ Unfortunately, migration barriers were merely computed for NaTiS_2_-3*R*_2_ (0.19 eV)^26^ and a hypothetical Na_*x*_TiS_2_ with O1 coordination/layer sequence (1.02 eV).^27^ Trigonal-antiprismatic coordination, as realised in the polytype 3*R*_2_, seems to hinder diffusion, so that diffusivities and migration barriers should be lower and higher in it, respectively, than in the other polytypes.^28^

Our dealing with these materials is less inspired by prospective application as an ion conductor than by improving the grasp of ion diffusion in them and a comparing them to their lithium congeners. This is why, herein, we report on the revised crystal structure of and sodium-ion diffusion in Na_0.5_TiS_2_-3*R*_1_ based on high-temperature neutron diffraction (ND). The study comprises topological analyses, the visualisation of diffusion pathways using the probability-density function (PDF) and maps of the scattering-length density (SLD) reconstructed *via* maximum-entropy methods (MEM), as well as an evaluation of migration barriers using the effective one-particle potential (OPP). Results are complemented by temperature-dependent X-ray diffraction (XRD), elemental analyses, and comments on the related compound Na_0.9_TiS_2_-2*H*.

## Experimental

### Analytical method

Hydrogen and sulphur contents were determined using a “Thermo Finnigan Flash EA 1112” analyser. Sodium was determined *via* optical emission spectroscopy with an inductively coupled plasma (ICP-OES) using a “Thermo Fisher Scientific iCAP 6300 Duo” evaluating the emission line at 589.5 nm.

The numbers of titanium ions per formula unit (with respect to sodium) were determined *via* wavelength-dispersive X-ray fluorescence (XRF) measurements on a “PANalytical Axios” spectrometer evaluating the intensities for the Na-K_α_ and Ti-K_α_ transitions. Samples were prepared from *ca.* 100 mg of analyte and 100 mg wax (Hoechst wax) performing uniaxial pressing (PerkinElmer hydraulic hand-press). Intensity ratios and sodium intensities were calibrated against linear regressions for three different mixtures of Na_2_S and TiS_2_ with *R*(Na/Ti) = *N*(Na) ≈ 0.4, 0.7, and 1.0 to account for instability/correlation of the titanium signal.

### Synthetic procedures

#### Synthesis of TiS_2_-1*T*

Polycrystalline TiS_2_-1*T* was prepared *via* direct synthesis from titanium (*ca.* 3.5 g, 1.00 eq.; Merck) and sulphur (2.10–2.20 eq.; Merck) in an evacuated silica ampoule. In a chamber furnace, the ampoule was heated to 400 °C with 25 °C h^−1^ and held there for 40 h to achieve complete reaction. The temperature was then elevated to 600 °C and kept for 99 h, after which the oven was turned off to cool to room temperature. Residual sulphur was evaporated *in vacuo* at 160 °C. Identity and purity of the resulting greyish black powder with metallic lustre (typically *ca.* 8 g) were confirmed using XRD.

#### General synthesis of Na_*x*_TiS_2_

TiS_2_-1*T* (*ca.* 3.5 g, 1.00 eq.) and Na_2_S (0.25 or 0.45 eq.; Sigma-Aldrich, ≥97%) were ground together in an agate mortar. The mixture was placed in a corundum crucible, which was subsequently heated to 700 °C with a rate of 400 °C h^−1^ in a tube furnace under H_2_S atmosphere (99.5%, Air Liquide), kept at that temperature for 2 h, and then cooled to ambient temperature with a rate of 200 °C h^−1^. The product was removed in a dinitrogen counter-flow and stored under argon. The reaction afforded Na_*x*_TiS_2_ (yield: *ca.* 99%) as greyish black, microcrystalline powder with a metallic lustre for high sodium content.

Found: H, 1.0(1); Na, 8.8(1); S, 48(1); *N*(Ti) = 1.0(2). Calc. for Na_0.5_S_2_Ti·½H_2_O: H, 0.8; Na, 8.7; S, 48.4%; *N*(Ti) = 1.0.

### X-ray diffraction

Measurements for phase identification and Rietveld refinement were carried out at ambient temperature on a “PANalytical X'Pert PRO MPD” diffractometer equipped with a “PIXcel” detector using nickel-filtered Cu-K_α_ radiation in Bragg–Brentano (*θ*–*θ*) geometry (see Fig. S1† for details). Temperature-dependent diffractograms were recorded in dinitrogen atmosphere on a “Rigaku SmartLab 3 kW” system using nickel-filtered Cu-K_α_ radiation.

### Neutron diffraction

Measurement was carried out at Institut Laue-Langevin (ILL) using the high-resolution two-axis diffractometer D2B with Ge(335)-monochromated constant-wavelength radiation (*λ* = 1.594 Å) in Debye–Scherrer geometry.^29,30^ Compacted powder samples were mounted in a vacuum high-temperature furnace inside a vanadium can (*d* = 13.9 mm, *h* = 49.8 mm). Measurements were carried out at ambient temperature, 300, 600, and 700 °C. Data were recorded with an array of 128 ^3^He tubes (height: 300 mm), yielding a final range of 0.10° ≤ 2*θ* ≤ 159.90° with Δ(2*θ*) = 0.05°. Initial Le-Bail fits and following Rietveld refinements were carried out using JANA2006.^31^ Neutron data were analytically corrected for absorption (cylindrical sample) and stripped of the irregular onset below 6.5° and, if necessary, cut-off reflections above 155°. Peak profiles were fitted with a pseudo-Voigt function using the Thompson–Cox–Hastings approach (Gaussian parameters *U*, *V*, and *W*; Lorentzian parameter *X*). A zero-shift correction and an asymmetry correction according to Howard were applied.^32^ The background was modelled using ten Legendre polynomials interpolating between manually defined points. Relatively weak reflections of a by-phase assuming the space-group type *Fm*3̄*m* (probably steel from the sample environment) were treated with an appropriate Le-Bail fit (separate profile parameters).

As a starting point for Rietveld refinement, an atomic model of Na_0.55_TiS_2_ was imported from the ICSD and adjusted to reflect the actual cell content and symmetry.^33^ Anisotropic displacement parameters were refined for all atoms except for Ti1 at 18 °C, the very small displacement of which had to be constrained to isotropy to yield a positive-definite value. For Na1 at 600 and 700 °C, anharmonic displacement was observed. Terms up to the fourth order were tested and only kept in refinement if they were significant (|*C*_*ijk*_| ≥ 3*σ*[*C*_*ijk*_]) and led to a significant drop in *R* values. Thus, two additional unique parameters *C*_111_(Na1) and *C*_333_(Na1) were refined. Towards the end of refinement, *z*(Na1) was found to be within a 3*σ* interval of ⅙ (coplanarity of sodium ions) and was fixed at this value, yielding slightly better residuals despite one free parameter less.

Structure graphics were produced using Diamond 4.5 and VESTA.^34,35^ Refinement results are summarized in Table 2. Diffraction datasets are available in the ILL repository.^36^ CSD 1942255 to 1942259 contain the supplementary crystallographic data for this paper; these data can be obtained free of charge from FIZ Karlsruhe *via*https://www.ccdc.cam.ac.uk/structures.

**Table 2:** Details of crystal structures and refinements on neutron diffraction data

Formula	Na_0.5_TiS_2_			
*M*_r_/g mol^−1^	123.48			
*θ*/°C	18	300	600	700
Space group	*R*3̄*m*			
*a*/Å	3.43840(8)	3.45878(5)	3.47973(6)	3.48717(7)
*c*/Å	21.0431(6)	21.2010(5)	21.3240(7)	21.3635(8)
*V*/Å^−3^	215.453(14)	219.651(9)	223.610(12)	224.983(14)
*Z*	3	3	3	3
Meas./obs.^a^ reflections	73/69	79/74	79/71	79/71
*R*_p_	0.0268	0.0195	0.0178	0.0177
w*R*_p_^b^	0.0359	0.0257	0.0232	0.0230
*R*_exp_	0.0176	0.0176	0.0175	0.0175
*R*_F_	0.0333	0.0320	0.0352	0.0414
*R*_I_ a	0.0520	0.0462	0.0438	0.0464

a *I* > 3*σ*(*I*).

b w = 1/[*σ*^2^(*I*) + (0.01*I*)^2^].

### Procrystal void analysis

Procrystal voids in the TiS_2_^*x*−^ substructures were analysed using CrystalExplorer 17.5 with default options.^37^ Models were based on the revised room-temperature structure of Na_0.5_TiS_2_-3*R*_1_ and structures of NaTiS_2_-2*H*, NaTiS_2_-3*R*_2_, and Na_0.3_TiS_2_-6*R* from the ICSD.^33^ Lower bounds for contiguous void networks were found by successively altering the procrystal density in steps of Δ*ρ*_pro_ = 0.0001 a.u.

### MEM reconstruction

Dysnomia 1.0 was used for MEM-reconstruction of SLDs from final structure factors as put out by JANA2006.^38^ The unit cell was divided into 96 × 96 × 588 voxels. Starting from a uniform intensity prior, the limited-memory Broyden–Fletcher–Goldfarb–Shanno (L-BFGS) algorithm^39^ was employed with uncertainties augmented by *E* = 0.5 and relative weights set to *λ*_2_ = 1, *λ*_*n*_ = 0 for *n* ≥ 4 to avoid overfitting. Final *R*_F_/w*R*_F_ were 0.0342/0.0305 and 0.0376/0.0328 for data acquired at 600 and 700 °C, respectively.

### OPP calculation

Sodium OPPs were calculated from PDFs as well as from MEM-reconstructed SLDs using CalcOPP 2.0.1.^40^ In the latter case, the maximal positive SLD found within the sodium layer was set to represent a potential energy of *V* = 0. For error estimation on PDF-derived data, the Monte-Carlo routines implemented in JANA2006 were employed (10 000 iterations, final accuracy < 1%).

## Results and discussion

### Synthesis and structure

The intercalates Na_*x*_TiS_2_ can be synthesized from the binary sulphides in a H_2_S feed. At 700 °C, *ca.* 3–4% of H_2_S decompose into H_2_ and S_*n*_.^41^ This creates a mildly reductive atmosphere that acts according to eqn (3).

3 *x*Na_2_S + 2TiS_2_ + *x*H_2_ → 2Na_*x*_TiS_2_ + *x*H_2_S

Compounds of this type are very hygroscopic and may reversibly co-intercalate up to two equivalents of water per formula unit without degradation.^42,43^ Elemental analyses show that even short handling in air is enough to effect absorption of *ca.* half an equivalent of water (no significant difference between stored samples with and without neutron diffractometry was observed). At the elevated temperatures examined herein, however, the samples dehydrate completely.

**Fig. 2:**
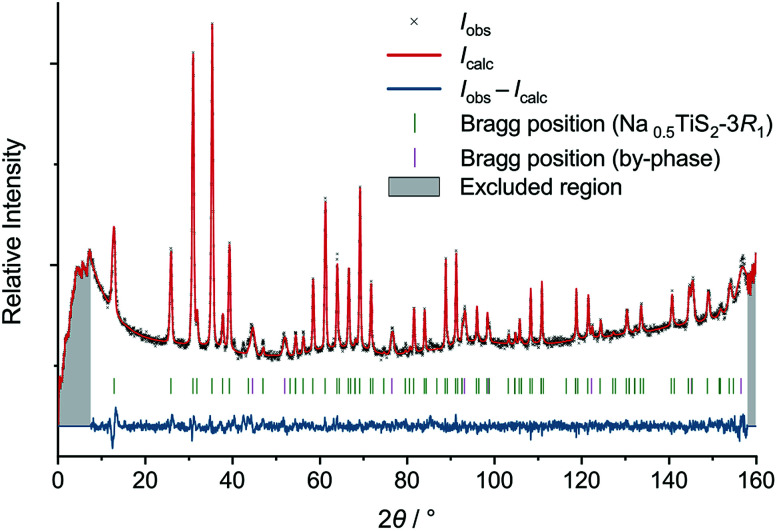
Neutron diffractogram of Na_0.5_TiS_2_-3*R*_1_ (revised structure) at 700 °C with results of Rietveld refinement.

During refinement of the initial model derived from Na_0.55_TiS_2_-3*R*_1_ against ND data, occupation of a second sodium position at (⅔, ⅓, ∼0.17), strong correlation of the sulphide ions' *z* coordinates, and refinement instability occurred. A closer look at the structures in the hitherto assigned space group *R*3*m* and its supergroup *R*3̄*m* reveals the following differences: the two unique sulphide positions with *z* = 0.39 and 0.60 in the former correspond to a single one with *z* = 0.40 in the latter (mismatch Δ*z* = 0.01). The two nearly equally occupied sodium positions with *z* = 0.17 correspond to a single position that has to be at *z* = ⅙ to warrant a coplanar arrangement (mismatch Δ*z* = 0.0). Refinement in *R*3̄*m* solved the initial problems (see Fig. 2 for exemplary and Fig. S4–S6† for remaining diffractograms). We found no hint at symmetry lowering at any temperature. While Na_0.55_TiS_2_ as synthesised by Rouxel *et al.*^6^ might represent an ordered variant, we assume a case of missed symmetry because of the following reasons: due to the perfect overlap of all reflections in *R*3*m* that are symmetry equivalent in *R*3̄*m*, only ever-so-subtle intensity differences distinguish one from the other (see Fig. S2†). For 3*R*_1_-isotypic K_0.6_VS_2_, Bronsema and Wiegers recognised the same problem and could not resolve it *via* X-ray powder diffraction.^44^ Furthermore, both sodium positions in *R*3*m* are crystal-chemically nearly identical and should not give rise to ordering. We thus conclude that the published structure is to be revised‡‡The previous assignment of the structure type “CuCrSe_2_–AgCrSe_2_(*R*3*m*)” to Na_0.55_TiS_2_-3*R*_1_ complicated a comparison to other structures: several isopointal but alloconfigurational structures were assigned this ill-defined type. It was split correctly in the course of this work (see Table S2†), the result being incorporated in the ICSD since May 2019.^13^.

**Fig. 3:**
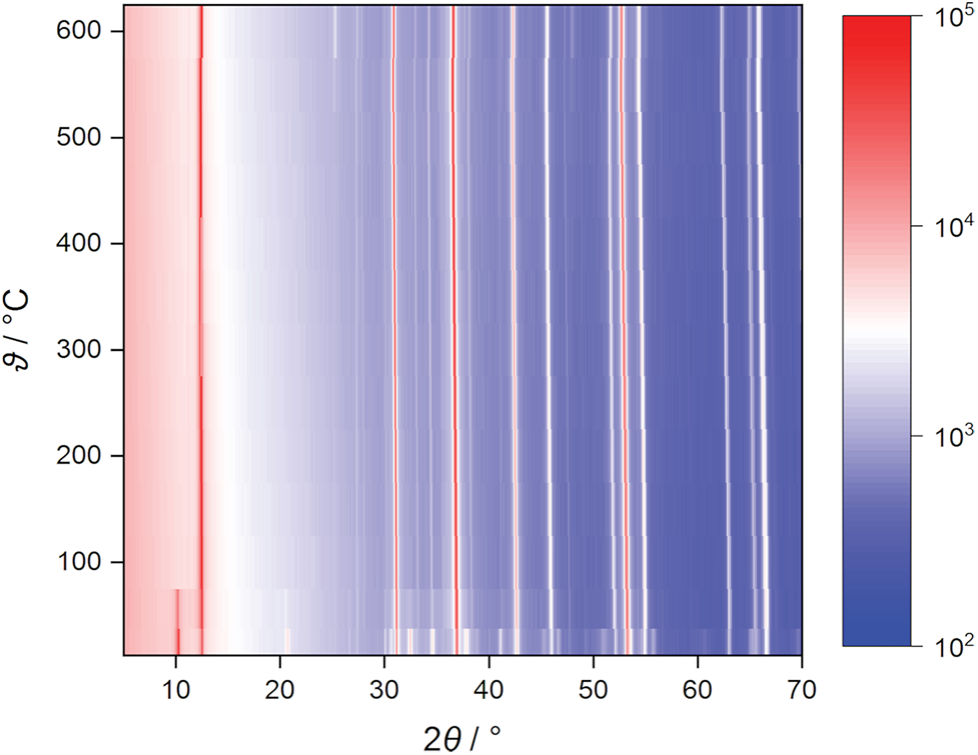
Heat map of uncorrected temperature-dependent X-ray diffractograms of Na_0.5_TiS_2_-3*R*_1_ in dinitrogen atmosphere. The colouring is based on raw counts; the scale is logarithmic.

Temperature-dependent XRD shows that, on heating, a previously stored Na_0.5_TiS_2_-3*R*_1_ sample loses co-intercalated water up to 100 °C (see Fig. 3, characteristic reflection at *ca.* 10°). No phase change akin to the 1*T*–3*R* transformation§§The compounds Li_*x*_TiS_2_-3*R* are isotypic to the high-alkali polytype Na_*x*_TiS_2_-3*R*_2_, not Na_*x*_TiS_2_-3*R*_1_. in the lighter homologue Li_0.7_TiS_2_ occurs.^45^ In dinitrogen atmosphere above 550 °C, decomposition starts (slight reflection-positional shift to higher angles, degradation of intensities, additional small reflections) under concomitant sulphur formation (found in the reaction chamber). While the absence of phase transitions is confirmed by ND, the sample has proven to be stable in a vanadium can surrounded by vacuum up to at least 700 °C. Cell dimensions lie in the range expected for *x* = 0.5 at ambient temperature and evolve in a roughly linear fashion during heating (*cf.* Fig. S7†).^46^ The latter also holds for the equivalent displacement parameters of the titanium and sulphide ions, whereas the one of the sodium ion starts at a value five to six times as large and grows disproportionately (see Fig. S8 and S9†). This is due to supposedly static disorder at room temperature and the onset of anharmonic dynamics (*i.e.*, ion diffusion) between 300 and 600 °C.

### Sodium diffusion pathways

We will describe the methods used herein very briefly. A more detailed overview has already been published.^47^ The pathways refer to thermally activated ion diffusion at temperatures high enough to warrant ample ion mobility.

#### Procrystal void analysis

The procrystal void analysis is a fast tool to find the most probable pathways for ion diffusion and to compare compounds, of which only the structure is known.^48^ Leaving out the mobile (in this case, sodium) ions, the method constructs the remaining static framework as a superposition of diffuse spherical electron densities centred at the atomic positions—the so-called procrystal density *ρ*_pro_. The latter contains voids that are characterised by an isovalue of a surface enclosing them: the lower the value, the “emptier” the void, the better its suitability for containing/conducting an ion.

**Fig. 4:**
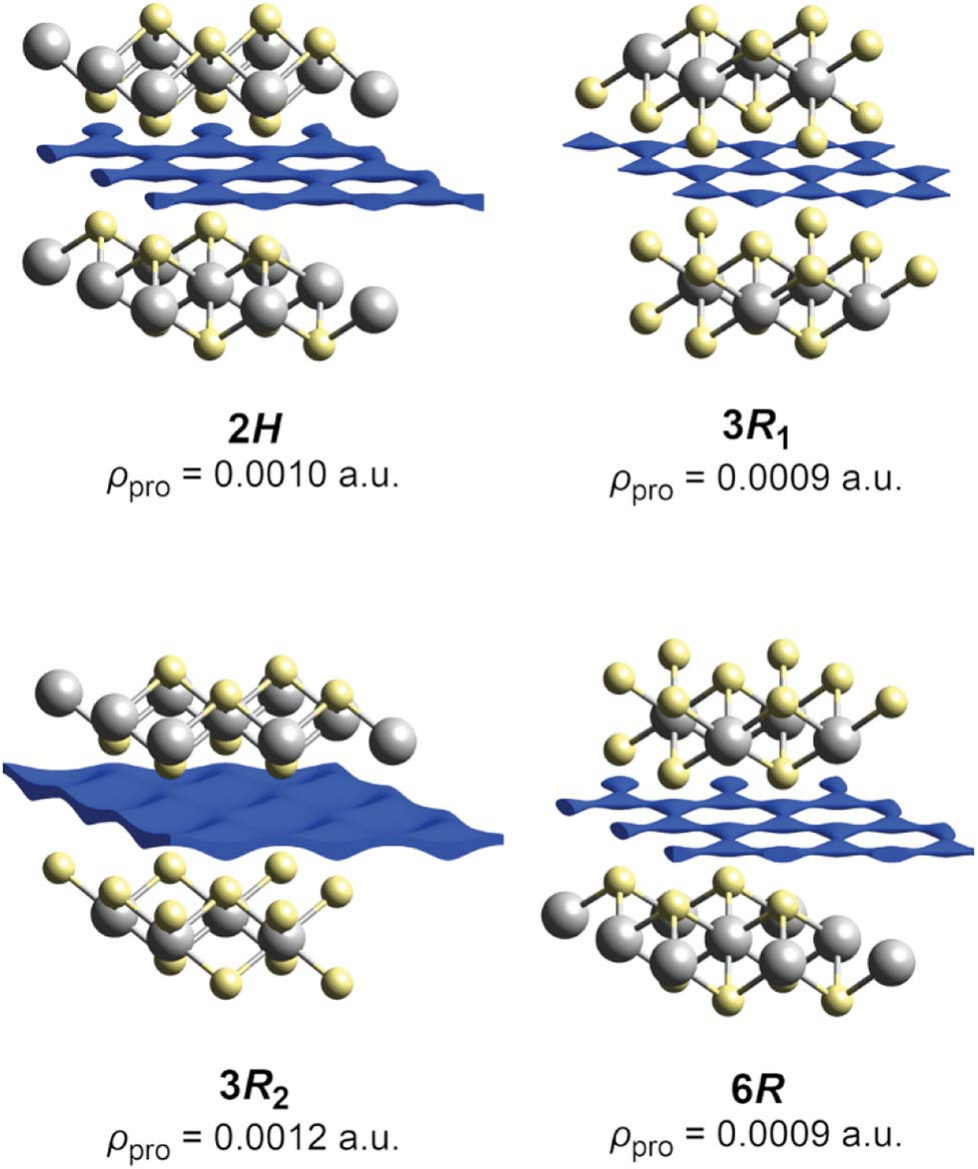
Procrystal void surfaces (blue) in the sodium layers of the TiS_2_^*x*−^ frameworks for different polytypes. Atoms are plotted with covalent radii (grey: titanium, yellow: sulphide ions). The depicted isovalue is the minimum for a contiguous surface.

The low and comparable isovalues, at which the voids in the polytypes 2*H*,¶¶*Caveat*: There had been a transcription error in the former ICSD record that was corrected in 2018. 3*R*_1_ (our revised structure), and 6*R* form contiguous paths, indicate that they are similarly well-suited sodium-ion conductors (see Fig. 4). The framework voids are located at the partly occupied sodium positions and connect to a honeycomb-like conduction pattern. The voids in the 3*R*_2_ structure also arrange in a honeycomb-like fashion and give no hint at any reason for an ordered occupation of half of them (like the current structural model suggests). The voids connect at a significantly higher isovalue to a carpet-like pattern, which suggests that this polytype is a worse and more isotropic sodium-ion conductor.

#### Probability-density function

The PDF of an atom at a point in space describes the probability of finding said atom displaced from its equilibrium position to said point. In the case of harmonic displacement, the depiction of the PDF as isosurface leads to the well-known spheres (isotropic) or ellipsoids (anisotropic). In the anharmonic case, however, forms that are more complex ensue. Because of this, a superposition of the PDFs of several atoms, called a joint probability-density function (JPDF), allows the visualisation of diffusion pathways.

**Fig. 5:**
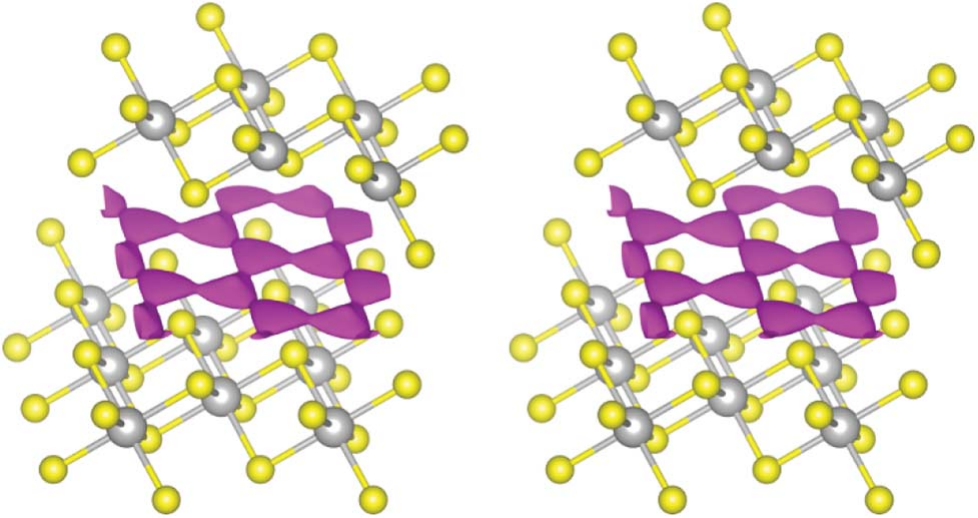
Joint sodium-PDF isosurface of 0.09 Å^−3^ (pink) in Na_0.5_TiS_2_-3*R*_1_ at 600 °C (left) and 700 °C (right). Atoms are plotted with covalent radii (grey: titanium, yellow: sulphide ions).

In the case at hand, the sodium ions are displaced along linear pathways between adjacent sodium positions at both relevant temperatures (*cf.* the similar isosurfaces in Fig. 5). In this way, the honeycomb-like conduction pattern predicted by procrystal void analysis is indeed experimentally reproduced.

#### MEM-reconstructed scattering-length density

**Fig. 6:**
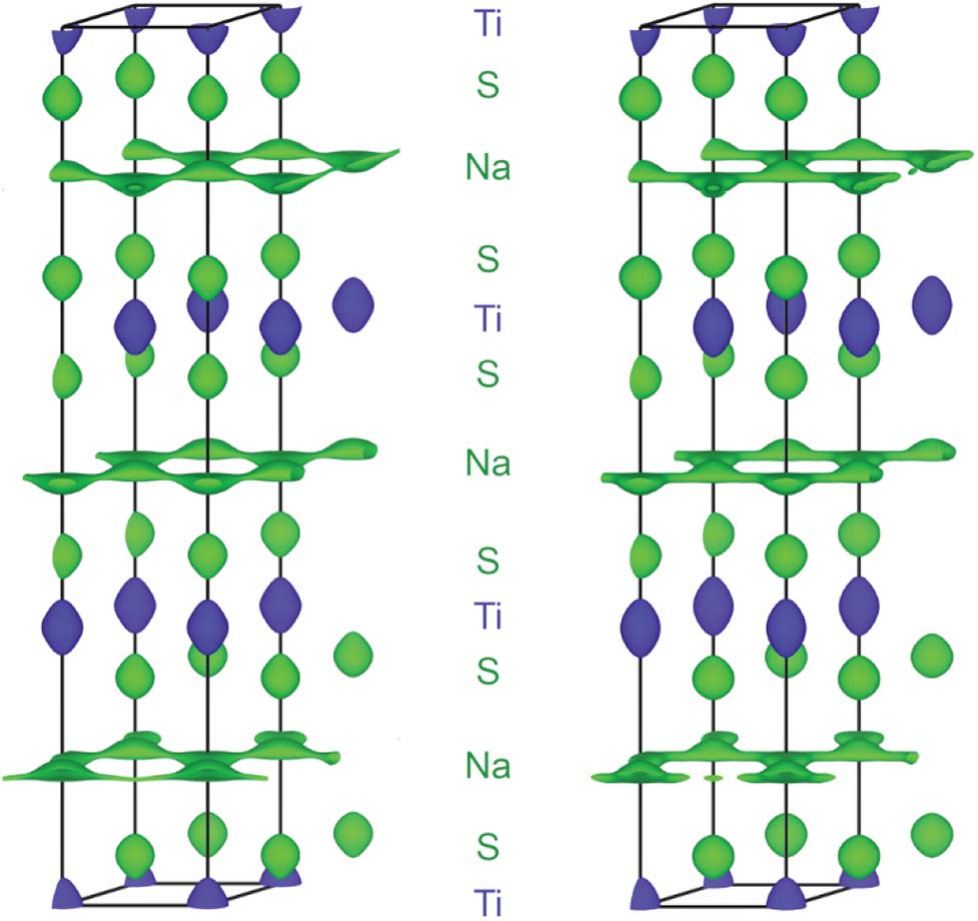
SLD isosurface of ±0.5 fm Å^−3^ (green: positive, blue: negative) in Na_0.5_TiS_2_-3*R*_1_ at 600 °C (left) and 700 °C (right) with view roughly along a*. Atomic layers are assigned to elements, unit cells are depicted in black.

To rule out bias, we have also inspected maps of the MEM-reconstructed SLD, which do not rely on modelling atomic displacement. In comparison to maps directly Fourier-transformed from the structure factors, they are deprived of artefacts and show a flatter distribution of noise. The maps show that our PDF model is indeed accurate and reproduces even finer features of the positive sodium-associated SLD (see Fig. 6).

In the lighter but more alkali-rich congeners Li_*x*_TiS_2_-3*R* (*x* = 0.7, 0.9), preferred ion pathways were found between adjacent lithium positions.^49^ Because of their ordering, however, they correspond to second-neighbours in disordered Na_0.5_TiS_2_-3*R*_1_.§ As a result, the pathways are not directly comparable. It is noteworthy that, in contrast to Li_*x*_TiS_2_-3*R*, we have not found a significant deviation from coplanarity. We attribute this to the differences in coordination polyhedra (trigonal antiprism *vs.* prism) and spatial arrangement (interlayer spacing larger by Δ*d* ≈ 0.96 Å, while ionic diameter only larger by 2Δ*r* = 0.52 Å (ref. 50) for the sodium compound).

### Migration barriers

For the estimation of the migration energy barrier associated with the found path, we calculated the OPP, which represents the energy landscape experienced by a mobile sodium ion (approximated as an Einstein oscillator that is subject to Boltzmann statistics at the classical limit). The activation energy of the migration (not comprising the defect formation energy) is the OPP at the bottleneck position, *i.e.*, the position of lowest probability along the pathway.

The calculation of OPPs from ND-derived PDFs is fairly established,^51^ but direct derivations from MEM-reconstructed SLDs without any further refinement are scarce.^52^ The latter is only warranted if an SLD shares basic characteristics of a probability density. Mathematically speaking, a PDF has to be non-negative, Lebesgue-integrable, and normalized to an integral of unity over the whole space. To give a reason for using the direct approach nonetheless, the following points address these issues not in a rigorous but hand-waving way:

• The OPP calculation deals with ratios of input values. Only the non-negative ones are taken into account.

• Numerically sampled SLDs are Lebesgue-integrable.

• For ratios of input values, normalization is unnecessary (the normalizing constant cancels).

Furthermore, the adequacy of the results is ensured by only taking the SLD solely caused by sodium ions (*i.e.*, within the sodium layers) into account.

In agreement with crystal-chemical assumptions, we found the bottleneck of migration at ⅓, ⅙, ⅙—in the middle of a straight line between adjacent sodium positions. The OPPs at this point are summarized in Table 3. Generally, the activation energy does not depend on the temperature as long as only a single migration mechanism is concerned and the structure is stable. For this method, however, this holds only if diffusion is fully activated because the migrating ions themselves are the probe for the potential. This is why slightly lower activation barriers are often found for higher temperatures—as is the case here for the central values—and are thought to be more reliable. Nevertheless, the PDF-derived energies are equal within 1*σ*. Currently, there is no computational way to estimate the errors on SLD-derived values. Judging by the uncertainties of the PDF-derived barriers, however, the central values are significantly lower, but of the same magnitude. As this is the first study employing both methods, it remains yet unknown if the deviation is systematic. It is, *e.g.*, possible that a restricted set of parameters models parts of the SLD imperfectly (thereby modelling a too low probability density resulting in a too high OPP) or that the SLD-derived OPP carries a considerable error.

**Table 3:** Activation energies (in eV) of sodium-ion migration in Na_0.5_TiS_2_-3*R*_1_

Calculation	At 600 °C	At 700 °C
From displacement-derived PDF	0.114(7)	0.108(8)
From MEM-reconstructed SLD	0.086	0.070

**Fig. 7:**
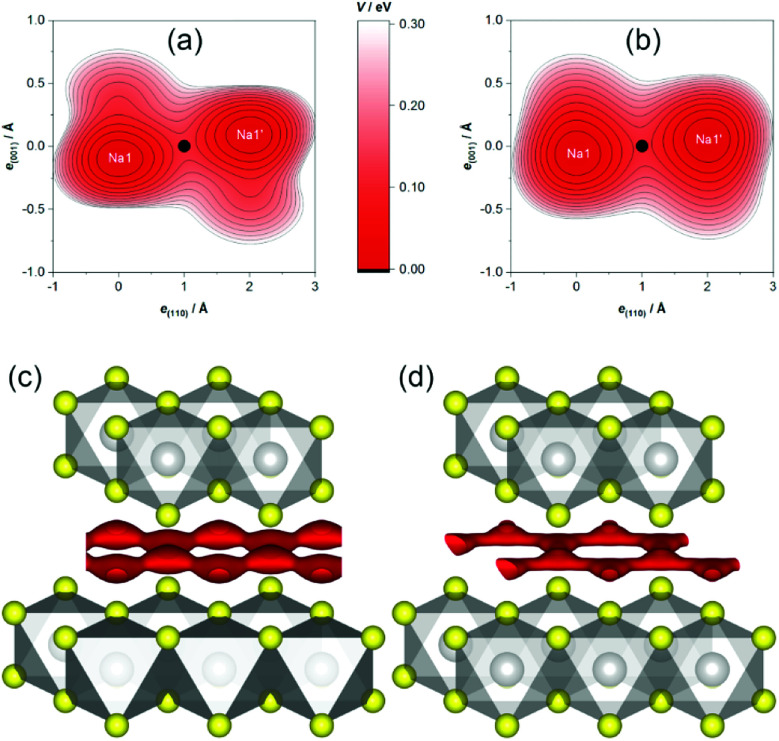
Sodium OPP *V* in Na_0.5_TiS_2_-3*R*_1_. Top: Contour plot of PDF-derived OPP within a plane containing two adjacent sodium ions at (a) 600 °C and (b) 700 °C (origin at Na1, bottleneck as black dot, contours: from *V*_0_ = 0 on with Δ*V* = 0.025 eV). Bottom: Isosurface plots of (c) PDF-derived and (d) SLD-derived OPP of *V* = 0.15 eV at 700 °C (atoms as with covalent radii; grey: titanium, yellow: sulphide ions, red: OPP isosurface).

The topography of the OPP landscape, on the other hand, is similar irrespective of temperature or calculation method (see Fig. 7). As temperature rises, the potential becomes flatter and gives access to a larger area (*cf.*Fig. 7a/b), but the basic shape is preserved. As the sodium PDF reproduces the features of the SLD (*vide supra*), the landscapes derived from both resemble each other (*cf.*Fig. 7c/d). The smaller volume enclosed by the same isosurface, however, shows that the SLD-derived OPP is steeper, therefore stemming from a more localised distribution.

The experimental barriers are lower than computed for defective NaTiS_2_-3*R*_2_ (0.19 eV)^26^ and measured for Li_0.7_TiS_2_-3*R* (*ca.* 0.5 eV).^49^ Procrystal void analysis already led us to expect that these homeotypic (by alkali occupation) structures be detrimental to alkali-ion conduction (*vide supra*). In addition, the lower number of vacancies (higher alkali content) in the latter hinders migration.^49^ For Na_0.5_TiS_2_-3*R*_1_, low activation barriers corroborate its role as a good sodium-ion conductor. Although a model all-solid-state sodium-ion battery using a Na_*x*_TiS_2_ electrode has been realized,^53^ problems with reactivity, phase transformations upon de-/intercalation, and mediocre cyclability seem to prohibit its effective use therein.^54^

### Remarks on “Na_0.9_TiS_2_-2*H*”

Our endeavour to explore also the more sodium-rich Na_0.9_TiS_2_-2*H* was hindered by synthesis problems. While XRD for phase identification and ND seemed to indicate a single-phase product of the desired polytype, closer inspection (see Fig. S3†) revealed at least two similar phases, possibly with a more complex (OD) structure. Because of the broader reflections in ND, we found no way of separating the contributions of different phases. A refined (unphysical) single-phase model averaging over all phases was at odds with elemental analysis: The former found only *ca.* 0.5 sodium ions per formula unit at the crystallographic positions (no significant positive residual SLD) while the latter conformed to the target composition.

Furthermore, we found a strong preferred orientation in (001) that agrees well with the plate-like visual appearance of the crystallites in the sample. In a temperature-dependent XRD experiment, we observed the 2*H*-like structure over the whole stability range. No signs of a transformation into a supposedly stable 3*R*_2_-like structure showed above 300 °C, even when heating for a longer time.

## Conclusions

We synthesised the intercalate Na_0.5_TiS_2_-3*R*_1_ from sodium and titanium sulphide in a mildly reducing H_2_S atmosphere. Our ND and XRD experiments showed that its structure, as reported in the literature, has to be revised: Na_0.5_TiS_2_-3*R*_1_ assumes the space-group type *R*3̄*m* (not *R*3*m*) with disordered (not ordered) sodium ions and one (not two) crystallographically independent sulphide position. Up to *ca.* 100 °C, the compound reversibly co-intercalates half an equivalent of water. It is stable with respect to phase transformations up to its decomposition temperature.

Sodium-ion diffusion manifests in anharmonic displacement at 600 and 700 °C. In agreement with expectations from topological analysis of the static TiS_2_^*x*−^ framework, maps of the MEM-reconstructed SLD and modelled PDFs show a honeycomb-like conduction pattern with linear almost in-plane pathways between adjacent (partly occupied) sodium positions. The bottlenecks of migration are found at the midpoint of said pathways. The associated OPPs at 700 °C indicate low activation barriers of 0.108(8) eV or even 0.070 eV (derived from PDF or SLD, respectively).

Unfortunately, we could not complement these findings with data for Na_0.9_TiS_2_-2*H*. We presume that our synthesis method led to segregation into at least two 2*H*-like phases—one with lower, one with higher sodium content. Possibly, these are closely related to Na_2_TiS_3_ with its OD structure. Bouwmeester *et al.* had already found such by-phases (re-)forming during high- to mid-temperature syntheses *ex elementis* with high sodium load.^8^ The potential of arranging

• pure sodium layers (*e.g.*, in NaTiS_2_), their defective (*e.g.*, in Na_*x*_TiS_2_ with *x* < 1), titanium-mixed (*e.g.*, in Na_2_TiS_3_), or even as-well-as variants (*e.g.*, in Na_*x*_Ti_1+*δ*_S_2_ with *x* < 1 − 3*δ*)

• with their sodium, titanium, and vacancy positions ordered or unordered within one layer

• in an ordered or unordered stacking sequence

accounts for compelling structural complexity in layered sodium titanium sulphides. This is particularly pronounced at the fringes of very high or very low alkali content and makes further exploration, especially of the interplay between sodium mobility and structural disorder, worthwhile.

## Conflicts of interest

There are no conflicts to declare.

## Supplementary Material

RA-009-C9RA05690D-s001

RA-009-C9RA05690D-s002
