# Supplementary material for: Structural complexities and sodium-ion diffusion in the intercalates NaxTiS2: move it, change it, re-diffract it
Source: RSC Adv. 2019 Sep 3;9(48):27780–8. doi: 10.1039/c9ra05690d (PMC9070861; doi:10.1039/c9ra05690d)
Supplement: RA-009-C9RA05690D-s001 [file RA-009-C9RA05690D-s001.pdf]

Electronic Supplementary Material (ESI) for RSC Advances.
This journal is © The Royal Society of Chemistry 2019

Structural complexities and sodium-ion diffusion in the intercalates Na_xTiS_2: move it, change it, re-diffract it

Electronic Supplementary Information (ESI)

Dennis Wiedemann, *[a] Emmanuelle Suard [b] and Martin Lerch [a]

[a]Technische Universität Berlin, Institut für Chemie, Berlin, 10623, Germany. E-mail: dennis.wiedemann@chem.tu-berlin.de
[b]Institut Laue-Langevin, Grenoble, 38042, France

Content

1 Details on X-ray iffraction 2
2 Details on neutron diffraction 4
3 ICSD orrections 7
4 References 7

1 Details on X-ray diffraction

Measurement was carried out at ambient temperature on a “PANalytical X’Pert PRO MPD” diffractometer equipped with a “PIXcel” detector using nickel-filtered Cu-K_{α} radiation (λ_1 = 1.54056 Å, λ_2 = 1.54439 Å, I_2/I_1 = 0.5) in Bragg–Brentano (θ–θ) geometry. Initial Le-Bail fits and following calculations were carried out using JANA2006.[1] Peak profiles were fitted with a pseudo-Voigt function using the Thompson–Cox–Hastings approach (Gaussian parameters *U*, *V*, and *W*; Lorentzian parameter *X*).[2] Asymmetry was corrected for using the Bérar–Baldinozzi method with four parameters.[3] Displacement and transparency corrections were refined. The background was modelled using 15 Legendre polynomials between manually defined points. A scale factor and two parameters for roughness correction according to Pitschke, Hermann, and Mattern were employed.[4] Atomic displacements were refined with an isotropic model.

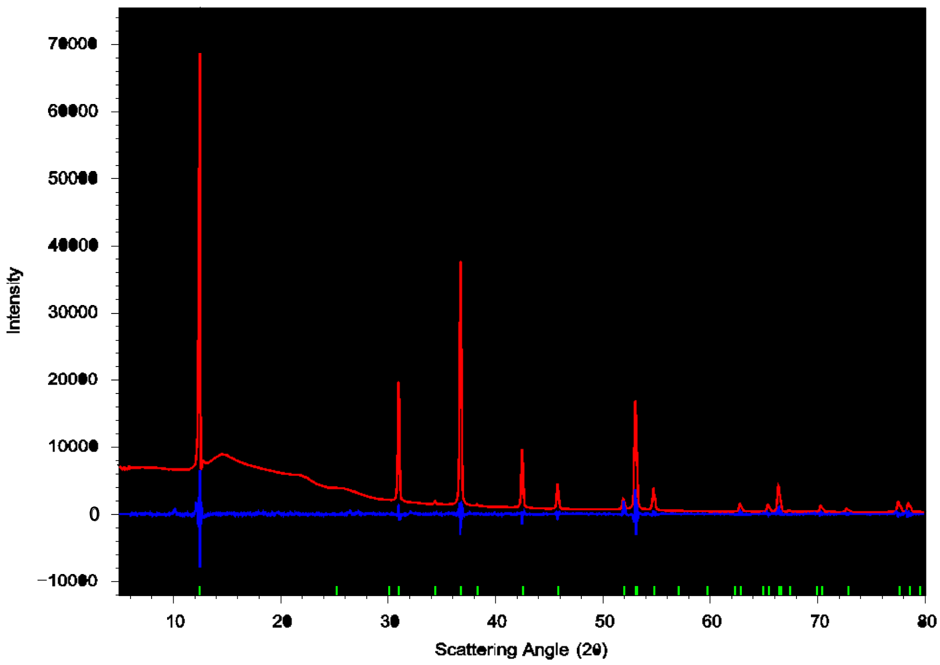

Fig. S1 X-ray diffractogram of $Na_{0.5}TiS_2$-3R_1 with results of Rietveld refinement (R_p = 0.0363, wR_p = 0.0621, R_{exp} = 0.0186, R_F = 0.0428, R_I = 0.0660). Red: calculated, black: observed, blue: difference density; green: Bragg positions.

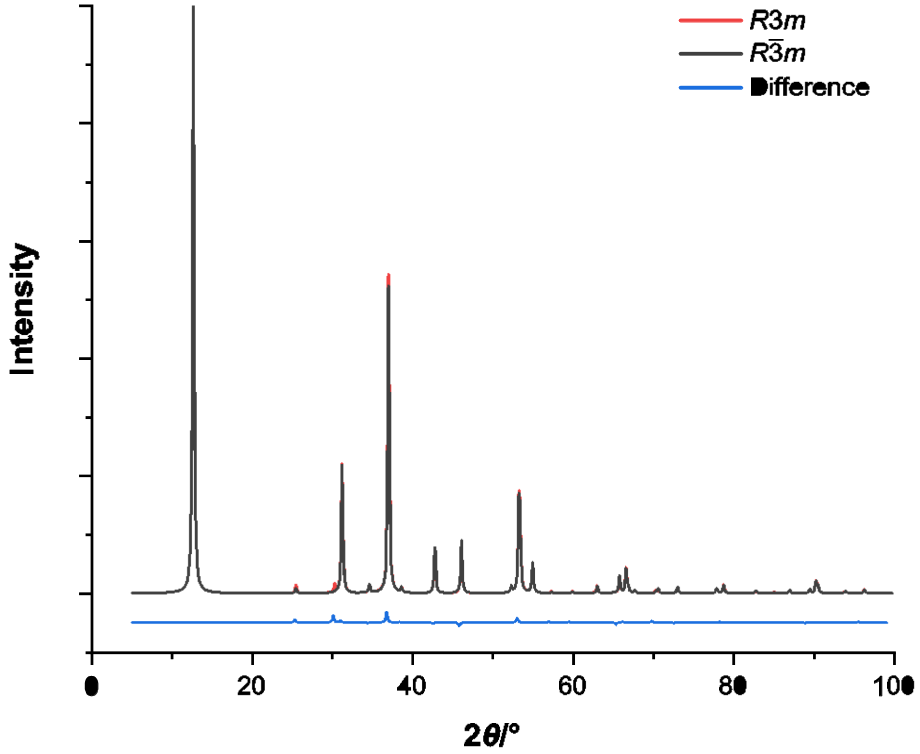

Fig. S2 Comparison of the X-ray diffractograms simulated for $Na_{0.55}TiS_2$-3R_1 in *R*3*m* (model from literature) and *R*3*m* (symmetrized by slightly adjusting atomic positions with disordered sodium position) with λ = 1.54056 Å (Cu-$K_{\alpha 1}$) and *f* = 0.25° (Gaussian profile).

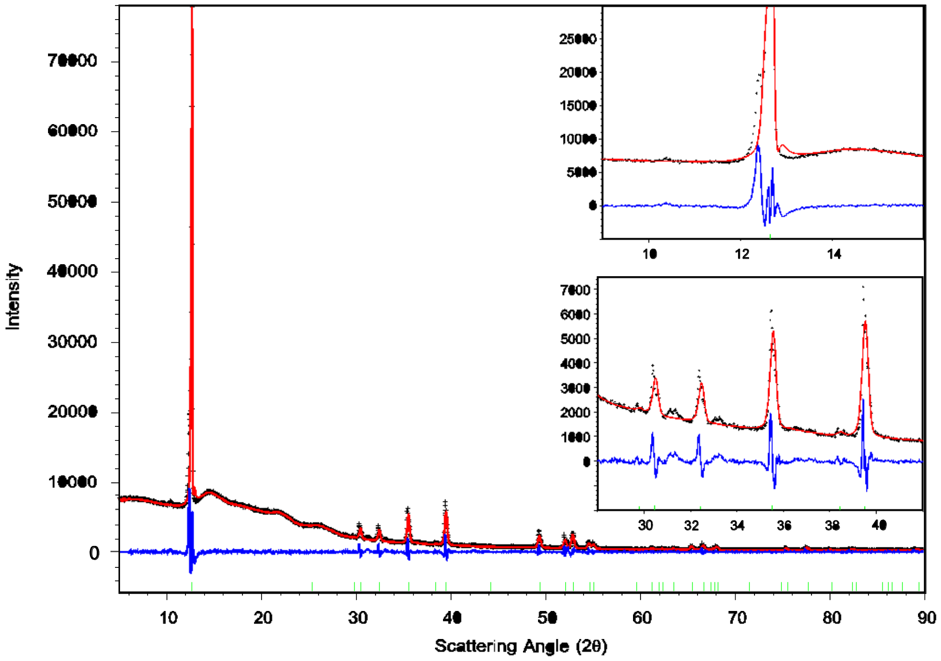

Fig. S3 X-ray diffractogram of "$Na_{0.9}TiS_2$-2*H*" with results of Le-Bail fit (R_p = 0.0408, wR_p = 0.0828). Red: calculated, black: observed, blue: difference density; green: Bragg positions. Insets show details of the low-angle regime. The high relative intensity of the first reflection indicates a strongly preferred orientation along (001). The low overall quality of the fit is due to the neglect of reflections (*e.g.*, a prominent shoulder at *ca.* 12.4°) or their features (*e.g.*, splitting at *ca.* 30.5, 32.5, 35.5, and 39.5°).

2 Details on neutron diffraction

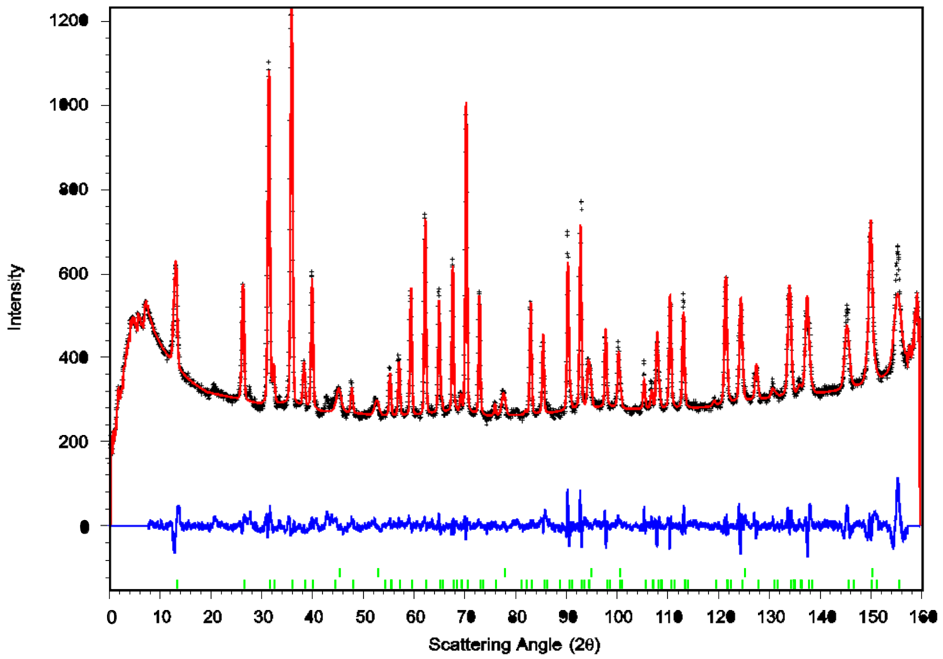

Fig. S4 Neutron diffractogram of $Na_{0.5}TiS_2$-3R_1 at 19 °C with results of Rietveld refinement. Red: calculated, black: observed, blue: difference density; green: Bragg positions for $Na_{0.5}TiS_2$-3R_1 (bottom) and *Fm*3*m* by-phase (top).

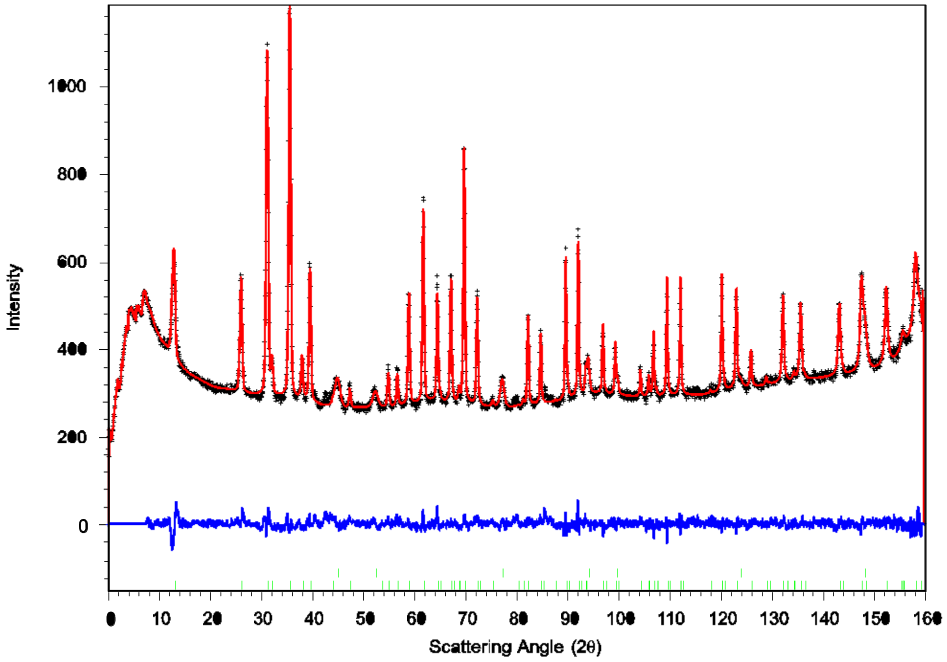

Fig. S5 Neutron diffractogram of $Na_{0.5}TiS_2$-3R_1 at 300 °C with results of Rietveld refinement. Red: calculated, black: observed, blue: difference density; green: Bragg positions for $Na_{0.5}TiS_2$-3R_1 (bottom) and *Fm*3*m* by-phase (top).

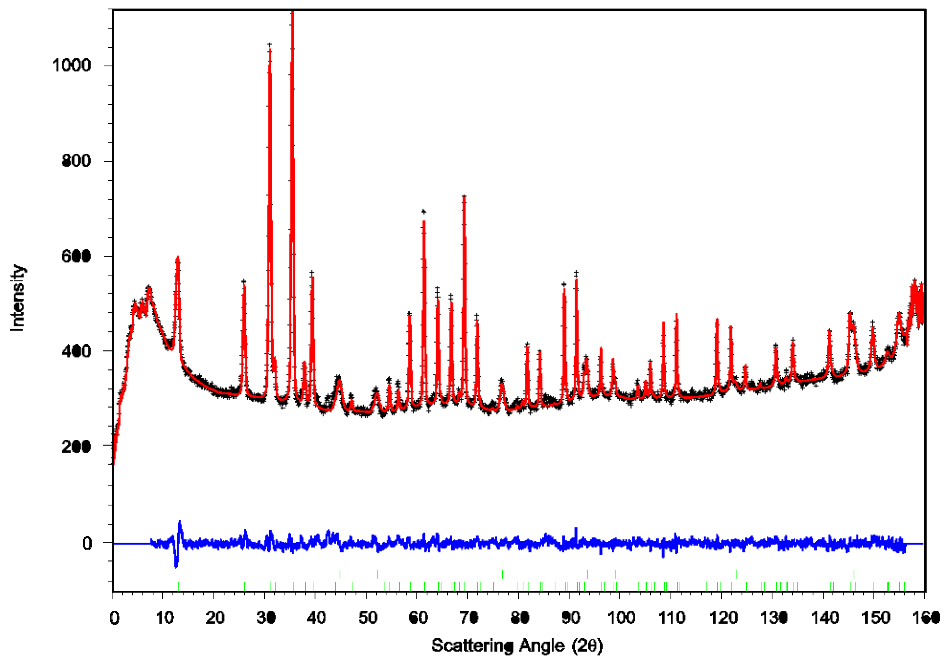

Fig. S6 Neutron diffractogram of $Na_{0.5}TiS_2$-3R_1 at 600 °C with results of Rietveld refinement. Red: calculated, black: observed, blue: difference density; green: Bragg positions for $Na_{0.5}TiS_2$-3R_1 (bottom) and *Fm*3*m* by-phase (top).

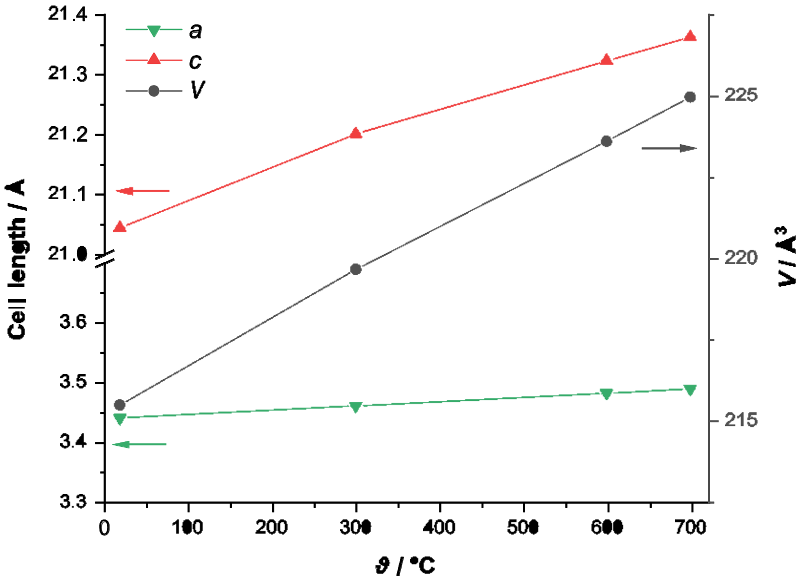

Fig. S7 Temperature evolution of cell lengths and volume in $Na_{0.5}TiS_2$-3R_1. Lines are merely guides to the eye; error bars are smaller than symbols.

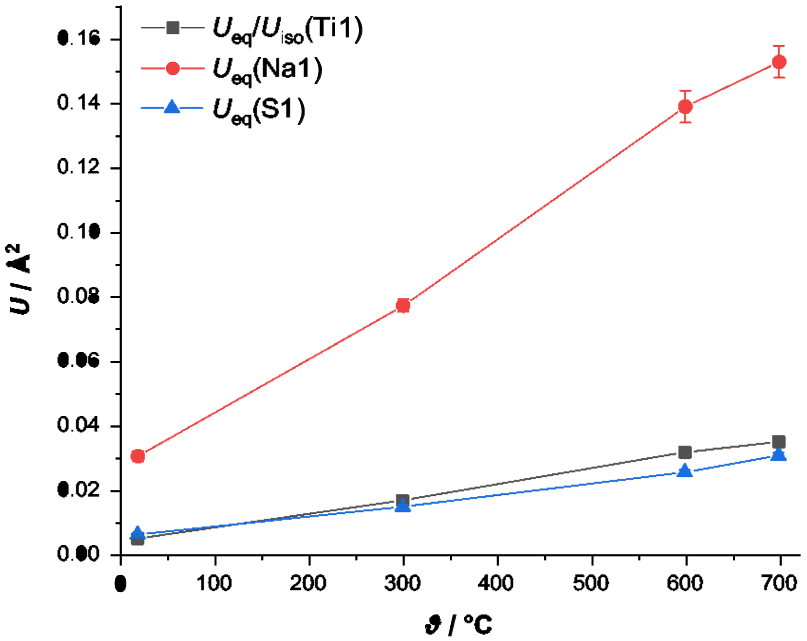

Fig. S8 Temperature evolution of equivalent displacement parameters in $Na_{0.5}TiS_2$-3R_1. Lines are merely guides to the eye; error bars are generally smaller than symbols.

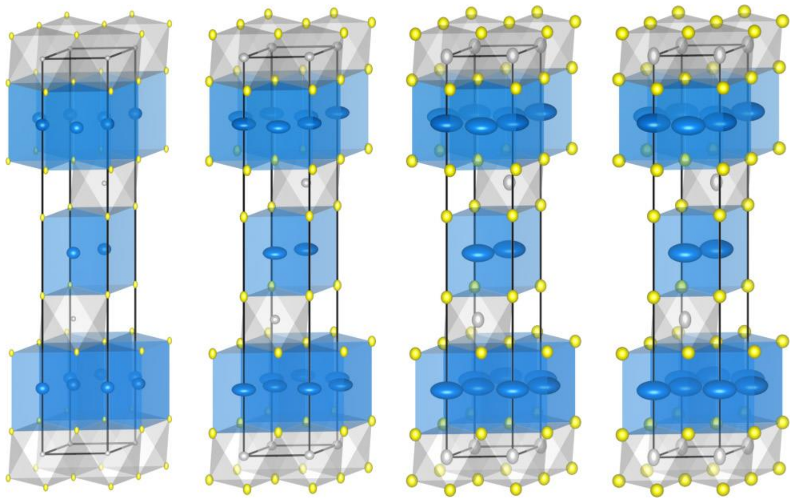

Fig. S9 Crystal structure of $Na_{0.5}TiS_2$-3R_1 at 18, 300, 600, and 700 °C (from left to right) according to neutron diffraction. Atoms as ellipsoids of 75% probability (harmonic displacement only); grey: titanium, blue: sodium, yellow: sulphide ions; unit cell in black.

3 ICSD Corrections

Table S1. Reassignment of isopointal structures with the formerly assigned type “$CuCrSe_2$-$AgCrSe_2$($R3m$)” to alloconfigurational structure types (effective since May 2019).

ICSD Number	Structured Formula	Structure Type
1884	InSe	γ-InSe
2308	InSe	γ-InSe
9992	$Na_{0.6}CoO_2$	—
23002	InSe	γ-InSe
23448	$KSnS_2$	$CuCrSe_2$
23449	$RbSnS_2$	$CuCrSe_2$
24796	$CuCrS_2$	$CuCrSe_2$
24797	$AgCrS_2$	$CuCrSe_2$
24798	$CuCrSe_2$	$CuCrSe_2$
24799	$AgCrSe_2$	$CuCrSe_2$
25625	$CuCrSe_2$	$CuCrSe_2$
25626	$AgCrSe_2$	$AgCrS_2$
25627	$CuCrS_2$	$AgCrS_2$
25628	$AgCrS_2$	$AgCrS_2$
40819	$K_{0.6}VS_2$	$CuCrSe_2$
41477	InSe	γ-InSe
42393	$CuCrS_2$	$CuCrSe_2$
42394	$CuCrS_2$	$CuCrSe_2$
42395	$AgCrS_2$	$CuCrSe_2$
42396	$AgCrS_2$	$CuCrSe_2$
42397	$AgCrSe_2$	$CuCrSe_2$
42398	$AgCrSe_2$	$CuCrSe_2$
68423	$AgCrSe_2$	$CuCrSe_2$
71092	$Na_{0.55}TiS_2$	$CuCrSe_2$
71932	$LiMoN_2$	$AgCrS_2$
73388	GaSe	γ-InSe
76540	$Na_{0.6}(Ti_{0.4}V_{0.6})S_2$	$CuCrSe_2$
76542	$Na_{0.5}VS_2$	—
76550	$Na_{0.6}VSe_2$	$CuCrSe_2$
77596	$Na_{0.6}VSe_2$	$CuCrSe_2$
77990	$RbTiS_2$	$CuCrSe_2$
84639	$CrWN_2$	$AgCrS_2$
88852	$AuCrS_2$	$AgCrS_2$
89454	$(H_{0.19}Na_{0.06}K_{0.25})CoO_2 \cdot 0.3\,H_2O$	—
100124	$Na_{0.6}VSe_2$	—
100594	$CuCrS_2$	$CuCrSe_2$
100595	$Cu_{1.016}Cr_{0.91}S_2$	—
161375	$Na_{0.62}CoO_2$	—
184736	$Na_{0.8}(Ni_{0.33}Mn_{0.33}Co_{0.33})O_2$	$CuCrSe_2$
187401	$CuCrSe_2$	$CuCrSe_2$
200983	$Na_{0.7}(Cr_{0.7}Ti_{0.3})S_2$	$CuCrSe_2$
201396	$TlCrS_2$	$CuCrSe_2$
291155	$Na_{0.5796}(Mn_{0.65}Co_{0.18}Ni_{0.17})O_2$	$CuCrSe_2$
604972	$(Ag_{0.5}Cu_{0.5})CrS_2$	$CuCrSe_2$
604981	$AgCrS_2$	$CuCrSe_2$
604993	$AgCrSe_2$	$CuCrSe_2$
605002	$AgCrTe_2$	$CuCrSe_2$
605616	$AgNiSe_2$	$CuCrSe_2$
605619	$AgNiTe_2$	$CuCrSe_2$
625764	$CuCrS_2$	$CuCrSe_2$
625799	$CuCrSe_2$	$CuCrSe_2$
626736	$TlCrSe_2$	$AgCrS_2$
629005	$(CuSb)Te_2$	γ-InSe
639178	$HgTaS_2$	—
640479	InSe	γ-InSe
640483	InSe	γ-InSe
640505	InSe	γ-InSe
640507	InSe	γ-InSe
641335	$KTiS_2$	$CuCrSe_2$
644994	$NaVS_2$	—

4 References

1 V. Petříček, M. Dušek and L. Palatinus, *Z. Kristallogr. – Cryst. Mater.*, 2014, **229**, 345–352.

2 P. Thompson, D. E. Cox and J. B. Hastings, *J. Appl. Crystallogr.*, 1987, **20**, 79–83.

3 J.-F. Bérar and G. Baldinozzi, *J. Appl. Crystallogr.*, 1993, **26**, 128–129.

4 W. Pitschke, H. Hermann and N. Mattern, *Powder Diffr.*, 1993, **8**, 74–83.
